# Potentiating Lung Mucosal Immunity Through Intranasal Vaccination

**DOI:** 10.3389/fimmu.2021.808527

**Published:** 2021-12-14

**Authors:** Sean A. Nelson, Andrea J. Sant

**Affiliations:** David H. Smith Center for Vaccine Biology and Immunology, Department of Microbiology and Immunology, University of Rochester Medical Center, Rochester, NY, United States

**Keywords:** influenza, tissue resident memory, CD4 T cells, CD8 T cells, mucosal antibody response

## Abstract

Yearly administration of influenza vaccines is our best available tool for controlling influenza virus spread. However, both practical and immunological factors sometimes result in sub-optimal vaccine efficacy. The call for improved, or even universal, influenza vaccines within the field has led to development of pre-clinical and clinical vaccine candidates that aim to address limitations of current influenza vaccine approaches. Here, we consider the route of immunization as a critical factor in eliciting tissue resident memory (Trm) populations that are not a target of current licensed intramuscular vaccines. Intranasal vaccination has the potential to boost tissue resident B and T cell populations that reside within specific niches of the upper and lower respiratory tract. Within these niches, Trm cells are poised to respond rapidly to pathogen re-encounter by nature of their anatomic localization and their ability to rapidly deliver anti-pathogen effector functions. Unique features of mucosal immunity in the upper and lower respiratory tracts suggest that antigen localized to these regions is required for the elicitation of protective B and T cell immunity at these sites and will need to be considered as an important attribute of a rationally designed intranasal vaccine. Finally, we discuss outstanding questions and areas of future inquiry in the field of lung mucosal immunity.

## Current Influenza Vaccination Approaches

Annual vaccination represents the best currently available strategy to mitigate the disease burden caused by influenza virus infection (1, 2). Each year, influenza vaccines are updated to reflect antigenic changes that occurred in influenza viruses as a consequence of selective pressures to escape immune recognition (3). These vaccines may be formulated as split or subunit inactivated vaccines or recombinant HA-based vaccines that are administered at a peripheral site or through live attenuated influenza viruses (1). Live attenuated virus vaccines are administered intranasally and most commonly in children, although the efficacy of this approach for children has sometimes been challenged (4, 5).

Year-to-year efficacy of licensed influenza vaccines is highly variable, ranging from a historical low of 10% to as high as 60% in the U.S. (2). Influenza vaccine efficacy is affected by a wide range of factors, including aspects of influenza surveillance, basic immunology, and vaccine formulation. Because current influenza vaccines have elicited HA neutralizing antibodies as their primary aim, efficacy is highly dependent on accurate strain prediction and antigenic match between vaccine and circulating viruses (2). Even in cases where vaccine viruses are well matched to circulating viruses, two well-known immunological phenomena also contribute to variable vaccine efficacy. The recall of pre-existing immunity generated by infection and vaccination into subsequent responses, termed original antigenic sin, re-directs the immune response towards regions shared with the priming antigen and away from novel epitopes on related antigens (6). In contrast, B and T cells focus on a subset of antigenic determinants present on a given antigen, an adaptive immune phenomenon termed immunodominance, and affects which cells are drawn into the immune response to either infection or vaccination (7). Immunodominance of variable regions of the HA-head over more conserved sub-dominant regions like the HA-stalk interfere with protective immune responses targeting the conserved stalk region (6). This pattern of immunodominance was observed across diverse species ranging from mammals and birds to lampreys, suggesting that immunodominance of certain sites within HA is a consequence of protein-intrinsic properties (7). While subunit inactivated and recombinant vaccines have a favorable safety and cost profile, they suffer from reduced immunogenicity relative to older whole virus vaccines that contained more diverse viral proteins and innate immune agonists (8, 9). The low immunogenicity of current vaccine formulations are further compounded by non-adjuvanted inactivated vaccine formulations, which can reinforce immune sub-dominance of conserved regions of HA (6 **)**. Low intrinsic immunogenicity also necessitates yearly administration of influenza vaccines, which can blunt the vaccine responses in frequently vaccinated individuals (10–13). Live attenuated influenza vaccine formulations are only available to people in certain age demographics and their efficacy may be diminished by pre-existing immunity that blunts replication (1, 4, 5).

Despite significant investment in influenza vaccination efforts worldwide, we have not achieved adequate control of seasonal spread of influenza viruses with current vaccine approaches and remain vulnerable to emergence of novel strains with pandemic potential. “Universal” influenza virus vaccine candidates promise to address the shortcomings of current vaccine approaches by providing long-lasting immune responses against all influenza A and B lineages with stable protective efficacy across influenza seasons (14, 15). With many next-generation influenza vaccines currently in pre-clinical or clinical trials (16–18), we believe that the route of vaccination is a critical consideration for future influenza vaccine design efforts. Specifically targeting intranasal vaccination to boost immune cell populations localized to the respiratory tract microenvironment allows us to exploit the advantages of tissue resident memory cells relative to peripheral immune cells. Tissue resident memory cells that have been previously generated by infection or live attenuated virus vaccination possess a unique phenotype, effector functions, and localization that allows them to mediate rapid anti-pathogen responses and act as frontline defenders of the barrier sites in the respiratory tract (19). Here, we consider the merits of vaccination approaches that foster B and T cells responses localized to the respiratory tract as critical factors of vaccine design for respiratory tract vaccine candidates and describe the outstanding issues that hinder rational vaccine design efforts.

## Distinct Niches for Tissue Resident Memory Populations in the Respiratory Tract

Despite the contributions of both the local and systemic humoral response to protective immunity, current vaccine approaches only target the systemic humoral response. In cases where strain-specific systemic antibodies fail to neutralize virions and blunt infection, local immune cell populations in the respiratory tract can contribute to protection from infection. A unique subset of memory cells, termed tissue resident memory (Trm) cells, can reside in non-lymphoid tissues such as the respiratory tract independent of circulating populations of memory cells (20). Their positioning at mucosal sites where pathogens are encountered makes these Trm cells anatomically positioned to respond rapidly to pathogen re-encounter. In addition to their anatomical location, these Trm cells are functionally poised to respond rapidly to reinfection. Data from animal models and humans have highlighted that both B and T cells can assume tissue resident memory fates. Tissue resident memory cells can localize to at least two distinct anatomical sites within the respiratory tract: upper respiratory tract (URT) and lower respiratory tract (LRT). The nares, pharynx, and larynx comprise the URT, while the LRT consists of the trachea, bronchi, and lungs (21).

Within the URT and LRT, there are distinct niches that help to support the long-term persistence of memory cell subsets outside of secondary lymphoid organs. Nasal associated lymphoid tissues (NALT) consist of B cell areas surrounded by T cell areas in the URT, a site that cannot support naïve T cell priming and is primarily populated by memory B and T cells (21, 22). In contrast to the LRT, memory T cells in the URT can be recalled by local inflammation, can patrol the nasal turbinates, septum, and NALT, and may not wane as quickly as LRT-localized T cell populations **(** 23 **)**. Within the LRT, inducible bronchus-associated lymphoid tissues (iBALT) are generated in response to inflammatory stimuli following orchestrated interactions between stromal cells, professional APCs, and infiltrating B and T cells (24). An antigen-dependent niche is thought to be especially important for memory CD4 T cell responses and for the production of local antibodies (21). Repair associated memory deposits (RAMD) form around sites undergoing tissue regeneration following infection-induced injury of the bronchioles. While RAMD does not contain lymphoid-like structures like NALT or iBALT, RAMD is important for maintaining CD8 T cells at relatively high density during the resolution phase of infection (21). Finally, a combination of local proliferation and recruitment from the periphery contributes to maintenance of LRT memory cell populations. A subset of lung resident cells primed by infection emigrate from the lung and maintain their tissue resident memory program from the lung draining lymph node, thereby maintaining the ability to return to the lung *via* a process termed retrograde migration (25). This reverse migration to the lung may allow cells to be saved from gradual attrition of cells in the lung and be stably maintained independently of residual antigen (25). Despite this reservoir of cells in the draining lymph node, memory T cell populations in the LRT are known to undergo attrition over time likely as a consequence of imbalances between local cell death and replenishment by peripheral cells that home to the lung or local proliferation of cells within the LRT, as well as diminishing viral antigen persistence (26–28).

## Tissue Resident Memory CD4 and CD8 T Cell Responses

Memory T cell populations persisting at the site of infection have been shown to mediate protection from infection with distinct subtypes of influenza virus by targeting viral proteins conserved between viral isolates, a phenomenon termed heterosubtypic immunity (29–35). Both influenza-specific CD4 and CD8 T cells make critical contributions to anti-viral immune responses in the lung (36–39). CD4 T cells can produce anti-viral cytokines and kill infected cells through a perforin and granzyme dependent mechanism (40–42). CD4 T cells can promote innate cell responses in the lung and promote early activation of professional APCs through cytokine production (38, 43–46). CD4 T follicular helper cells can provide cognate help to influenza-specific B cells and CD8 T cells, including those that reside directly in the respiratory tract (47–52). CD8 T cells mediate cytotoxic killing of infected cells and secrete anti-viral effector cytokines (36–39). Effector CD8 T cells kill infected cells through Fas-FasL interactions and through perforin and granzyme mediated cytotoxicity (53, 54). Somewhat paradoxically, airway localized CD8 Trm cells display decreased cytotoxic function relative to effector CD8 T cells from secondary lymphoid organs (SLO). The microenvironment of the lung is thought to drive a distinct transcriptional and epigenetic profile relative to cells from SLO, resulting in decreased cytotoxicity (55). However, signaling through the IFNα receptor up-regulated GzmB protein expression in CD8 Trm cells, enhancing killing and control of early viral titers (54). In addition to killing functions, CD8 Trm cells isolated from the lung airways are able to produce effector cytokines more rapidly than cells isolated from SLO, producing IFNγ within two hours of antigen restimulation (54). When transferred to naïve recipients that were subsequently challenged with influenza virus, CD8 airway Trm cells reduced influenza virus copy number in the lung in an IFNγ dependent manner (56). In the absence of CD4 T cells, CD8 T cell memory formation and localization are impaired, demonstrating functional synergy between the two cell subsets (36–39). Thus, memory CD4 and CD8 T cells targeting conserved epitopes between viruses can mediate diverse, often synergistic antiviral effector functions and provide protection from infection independent of antibody.

Data from animal models of infection demonstrate enhanced protection afforded by tissue resident memory T cells relative to peripheral T cells, suggesting that localization of Trm populations is a critical factor underlying their protective potential. We showed that intranasal vaccination with an influenza NP-nanoparticle vaccine elicited a polyfunctional subset of CD4 T cells that persisted long-term in the lung. Adoptive transfer of CD4 T cells isolated from the lung of mice boosted with the influenza NP-nanoparticle vaccine was sufficient to mediate protection from a lethal influenza virus challenge (57). Others in the field have found that adoptive transfer of TCR transgenic CD4 Trm cells isolated from lung, but not spleen, also mediated protection from influenza challenge (58). Lung localized CD8 T cells primed by intranasal infection, but not intraperitoneal infection, contribute to heterosubtypic immunity and clearance of infectious virus while peripheral CD8 T cell populations make a minimal contribution to protection (35). SARS infection in mice could be controlled by an airway localized CD4 T cell population that produced IFNγ and facilitated help for respiratory dendritic cell migration and CD8 T cell priming (59). In the absence of B cells, adoptively transferred CD4 T cells can mediate perforin-dependent protection from influenza virus infection (40). A subset of CD4 T resident helper cells have been identified in the influenza infected lung that can provide help for lung localized CD8 Trm and B cell resident memory (Brm) responses (51, 52 **)**. CD4 resident helper cells co-localize with B cells in lymphoid structures of BALT, where they can persist long term (51, 52). T resident helper cells, like other Trm cell subsets, are dependent on sustained interactions with MHC-II in the lung. Inducible deletion of Bcl6 among CD4 T cells resulted in less co-localization of CD4 T cells and B cells within BALT and an overall decreased influenza-specific ASC recall response in the lung (52). Using the same Bcl6 depletion approach, others have implicated T resident helper cells in the generation of optimal CD8 Trm and Brm responses (51). Therefore by emphasizing only systemic antibody responses against HA, current vaccination strategies fail to boost respiratory tract Trm populations and exploit their enhanced protective potential relative to peripheral immune populations.

## Defining the Contributions of Local and Systemic Antibody Responses

While current findings provide strong support for the role of T cell Trm in protection of the respiratory tract from infection, B cells can also adopt a tissue resident memory fate and reside in non-lymphoid tissues. Findings from animal models suggest that memory B cells primed by infection seed the NALT, airway draining LNs, spleen, and lung, and that these cells maintain the potential to assume ASC fate upon stimulation. IgA isotype switched ASCs were maintained in the URT at least 8 weeks post infection, while the lung contained both IgA and IgG isotype switched ASCs (22). Detailed clonal analysis of the B cell responses to influenza infection also demonstrated that infection elicits a widely disseminated B cell response (60). This clonally diverse response includes a subset of influenza-specific B memory cells generated in SLO that migrate to the lung and become resident memory cells (60). These data suggest that while there is relatively stable output of plasmablasts and memory B cells from the germinal center reaction, there is a kinetic window for establishing tissue resident memory in the lung. B cells generated in the lung draining lymph node can home to the lung up to 28 days post infection and cells derived from the spleen can home to the lung for up to 14 days post infection (60). Others have shown that Brm is durable, persisting for at least 5 months post infection (61). When transferred into an immunodeficient host, lung B cells maintained the potential to differentiate into plasma cells that produced neutralizing antibodies within the lung. The production of local IgA and IgG was correlated with decreased viral spread (61). Local antigen within the lung is necessary for Brm formation and for lung resident B cells that are present to lead to an accelerated antibody secreting cell response in the lung following secondary challenge (62).

Current influenza virus vaccine approaches are designed to induce systemic IgG responses, but not local antibody responses in the respiratory mucosa. Given that the primary target of influenza virus infection is the epithelium that lines the respiratory tract, mucosal antibody responses in the respiratory tract are critical for preventing influenza virus infection (63). Data from humans and pre-clinical animal models of influenza virus infection have highlighted important differences in the mechanisms underlying antibody-mediated protection in the URT relative to the LRT, suggesting that a “one size fits all” vaccine approach may not be sufficient to elicit antibodies at both sites. An increased understanding of the route of natural infection with respiratory viruses in humans has highlighted that a wide size range of particles shed from the respiratory tract can carry infectious droplets, and that larger particles tend to deposit in the URT, while smaller particles can penetrate deeper into the lung reaching the LRT (64). Thus, depending on the properties of the inhaled viral particles, local URT or LRT antibody titers may be insufficient to blunt viral replication.

Mucosal IgA and IgM responses are responsible predominantly for protection in the URT, while protection in the LRT is mediated by transport of serum IgG (65, 66). There is also evidence of mucosal IgD antibodies in the URT, but their function remains relatively poorly understood (66). Antibodies arrive in the URT through a process of active transport of IgA and IgM isotypes produced by plasma cells in the lamina propria adjacent to the epithelial cell barrier by the polymeric Ig receptor (63). An extracellular component of the polymeric immunoglobulin receptor (pIgR), termed the secretory component, gives secretory IgA increased resistance to proteases and aids in release of antibody complexes transported through epithelial cells (63). Antibody transport from serum into the LRT, however, is mediated by the neonatal FC receptor (67, 68). IgA is known to exist in multiple forms within the respiratory mucosa, ranging from monomers to tetramers, and potentially even higher order polymeric structures with greater than 4 IgA subunits. Collection of nasal washes from human donors demonstrated that polymeric and tetrameric forms of IgA had the highest neutralization potential, and that neutralization potential decreased as immunoglobulin structure became smaller (69). The higher avidity of multimeric IgA complexes increases the breadth of sIgA reactivity relative to IgG, especially when antibody affinity for the target antigen is limiting (70, 71). Human vaccine trials targeting intranasal vaccination with existing IIV and WIV formulations boosted serum and mucosal antibody titers when virus neutralization in the URT was correlated with local IgA titers and serum neutralization was correlated with IgG titers (69, 72). Recent findings from the acute response to a COVID-19 infection shows that early plasmablasts elicited by infection are predominantly of an IgA isotype and possess a mucosal homing phenotype, and that secreted mucosal IgA was detected at higher titers than in matched serum samples (73). These findings suggest that by focusing on only eliciting serum antibody, current vaccine strategies are neglecting a critical aspect of the humoral response that provides protection for the URT.

## Considerations for Vaccine Approaches that Foster Respiratory Tract Localized Immunity

Future influenza vaccine development approaches must consider route of vaccination as a critical factor for vaccine efficacy. Different mucosal sites require different signals for establishment of Trm cells (20). Whereas Trm cells in the mucosa of the female reproductive tract can be recalled with inflammation alone, this “prime and pull” strategy does not elicit stable Trm cell populations in the lung (74). Both B and T cell populations in the lung require local antigen for establishment of Trm (21, 75–77). Thus, given the necessity for local antigen in the lung, intranasal vaccine approaches may show promise in establishing B and T cell memory within the human respiratory tract. In addition to the immunologic advantages of seeding immune cells directly at the site of infection, delivery of local antigen *via* intranasal vaccination presents the advantage of non-invasive needle free delivery, which may enhance vaccine compliance. Current vaccine approaches based on the master-donor LAIV backbone show promise in eliciting mucosal immune responses in naïve individuals but show a reduced efficacy in those with pre-existing memory, likely as a consequence of antibody blunting viral replication (4, 5). Approaches utilizing novel viral vectors to which humans lack pre-existing immunity or non-replicating antigens may increase efficacy (78). A number of pre-clinical and clinical vaccine candidates have shown tremendous promise in generating anti-influenza immune responses, including mucosal immune responses in the lung (79–81). Inclusion of adjuvants may enhance protective efficacy of non-replicating antigens. Although there is significant appeal for the addition of adjuvants, safety is a primary concern with the complex immunity that exists in human (82, 83). A novel polysaccharide-based adjuvant was found to be well tolerated in human phase I trials and displays a synergistic effect with other adjuvants, raising the possibility of its use as a safe but potent intranasal adjuvant candidate (84). Persistent antigen within the respiratory tract is required for the induction of long-lived Trm cell populations (21, 76). An adenoviral vector vaccine expressing influenza NP drove antigen expression for at least three months and resulted in stable Trm cell populations for at least one year (85). Inclusion of excipients may enhance antigen persistence at mucosal sites, as rapid clearance and antigen adsorption are likely to affect lung localized immune responses (69, 72). In addition to formulation considerations, the safety of eliciting a lung-localized immune response will need to be assessed in diverse human populations. The challenges of pre-existing memory and intrinsic immunogenicity introduced above, suggest that the choice of antigen in the immune response for next-generation vaccines will be critical. Approaches that overcome the immunodominance of HA by specifically targeting the HA stem (86–88), NA (89, 90), or highly conserved and immunodominant internal proteins like NP are excellent candidates to increase efficacy and breadth of protection (57, 91, 92). Evidence from humans suggest that the anti-HA stalk antibody repertoire may be enriched for polyreactivity, the ability to bind to multiple distinct antigens, thereby implicating the role of self-tolerance in shaping the antibody response to certain pathogens (93–97).

## Outstanding Questions in Lung Mucosal Immunity

While animal studies have been indispensable in the identification and characterization of B and T cell resident memory populations, the role of these tissue resident cell populations in human immunity to respiratory infections is understudied. Data from human tissue explants has identified robust populations of CD4 and CD8 Trm cells in human lung and defined a core transcriptional signature of human Trm (98, 99). Restimulation of CD8 T cells isolated from human lung has demonstrated that human CD8 lung Trm cells mediate many of the same effector functions associated with protection in mouse models, including cytotoxic degranulation, polyfunctional cytokine production, and proliferation in response to stimulation (100, 101). In a human challenge model of RSV infection, the frequency of virus specific CD8 T cells in the airway of the lung were correlated with lower symptom scores and viral load (102). Kinetics of the virus-specific T cell responses in the airway were distinct from the blood, highlighting difficulties in extrapolating the relationship between readily sampled peripheral blood and the immune cells at the site of infection (102). Further human challenge studies that sample the actively functioning respiratory tract will be critical in defining better correlates of protection and potentially lung-specific correlates of T cell mediated protection. Defining a lung-specific correlate of antibody-mediated protection may help address whether levels of local antibody are limiting. Repeated administration of current influenza vaccines elicits high serum antibody titers but antibody specificities or titers in the URT may be insufficient to provide complete protection from infection (103). Peripheral vaccination with weakly immunogenic vaccine formulations may also limit boosting of tissue resident T cell populations. In light of the findings from animal models demonstrating attrition of lung Trm, a greater understanding of the factors that underlie waning of immune cell populations, especially within the LRT, is necessary for any intranasal vaccination efforts aiming to provide durable protection (26–28). With safety as a primary focus, development of approved mucosal vaccine adjuvants may assist in intranasal vaccine design efforts (82).

## Data Availability Statement

The original contributions presented in the study are included in the article/supplementary material. Further inquiries can be directed to the corresponding author.

## Author Contributions

All authors listed have made a substantial, direct and intellectual contribution to the work, and approved it for publication.

## Funding

This work was supported by research grants from the NIAID Centers of Excellence for Influenza Research and Surveillance, the NIAID Collaborative Influenza Vaccine Innovation Centers, NIAID, and National Heart, Lung, and Blood Institute, including grants HHSN272201400005C, HHSN272201400005C-17A, HHSN272201400005C-16I, 75N93019C00052, R21AI145269, and 5T32HL066988-19.

## Conflict of Interest

The authors declare that the research was conducted in the absence of any commercial or financial relationships that could be construed as a potential conflict of interest.

## Publisher’s Note

All claims expressed in this article are solely those of the authors and do not necessarily represent those of their affiliated organizations, or those of the publisher, the editors and the reviewers. Any product that may be evaluated in this article, or claim that may be made by its manufacturer, is not guaranteed or endorsed by the publisher.
